# Advances in Next-Generation Immunotherapies for Ovarian Cancer: Mechanisms of Immune Evasion and Novel Therapeutic Targets

**DOI:** 10.3390/biom16020246

**Published:** 2026-02-04

**Authors:** Md Ataur Rahman, Maroua Jalouli, Mohammed Al-Zharani, Abdel Halim Harrath

**Affiliations:** 1 Department of Oncology, Karmanos Cancer Institute, Wayne State University, Detroit, MI 48201, USA; rahman23@wayne.edu; 2 Department of Biology, College of Science, Imam Mohammad Ibn Saud Islamic University (IMSIU), Riyadh 11623, Saudi Arabia; mejalouli@imamu.edu.sa (M.J.); mmylzahrani@imamu.edu.sa (M.A.-Z.); 3 Zoology Department, College of Science, King Saud University, Riyadh 11451, Saudi Arabia

**Keywords:** ovarian cancer, immunotherapy, immunosuppressive, immune evasion, tumor microenvironment, next-generation therapeutics

## Abstract

Ovarian cancer (OC) is a particularly lethal gynecological malignancy with few treatment options due to its late-stage diagnosis, extensive genetic heterogeneity, and frequent development of resistance to existing therapies. Immunotherapy has revolutionized the management and clinical outcome of numerous solid tumors, but its clinical benefit for OC has been limited, in part due to an extremely immunosuppressive tumor microenvironment (TME) and diverse, overlapping immune evasion mechanisms. In this review, we present a comprehensive and timely synthesis of next-generation immunotherapeutic approaches for ovarian cancer, emphasizing strategies that overcome the immunosuppressive tumor microenvironment and improve clinical responsiveness. We describe the emerging molecular mechanisms of immune evasion in OC, including altered antigen presentation, inhibition of T-cell activation (e.g., via immunological checkpoints, metabolic reprogramming), polarization of tumor-associated macrophages (TAMs), and dysfunction of natural killer (NK) cells. We also critically examine several emerging therapeutic approaches, including combination immune checkpoint blockade (ICB), bispecific T-cell engagers (BiTEs), neoantigen-based vaccines, chimeric antigen receptor (CAR)-T- and CAR-NK-cell therapies, oncolytic viruses (OVs), and nanoparticle-mediated immunomodulation. In addition, we highlight recent advances in tumor microenvironment–targeted therapies for ovarian cancer, focusing on strategies that modulate non-lymphoid components such as cancer-associated fibroblasts (CAFs), hypoxia-driven signaling, and the PI3K/AKT/mTOR axis to enhance antitumor immune responsiveness. Finally, we discuss how predictive biomarkers, multi-omics systems, and patient-derived organoid models are accelerating the development and deployment of precision immunotherapies for OC. We would like to highlight the translational promise of next-generation immunotherapies and identify novel molecular targets that may be leveraged to achieve durable responses in OC.

## 1. Introduction

Ovarian cancer (OC) remains the deadliest gynecologic cancer worldwide, with high mortality driven by non-specific symptoms, late-stage diagnosis and both innate and acquired resistance to current treatments [1,2]. Despite advances in cytoreductive surgery, platinum-based chemotherapy and molecularly targeted therapies, including PARP inhibitors and anti-angiogenic agents, long-term outcomes for patients with advanced-stage disease remain poor [3]. The 5-year overall survival has shown minimal improvement over the last few decades, and there is a need for new therapies to address the complex molecular and immune landscape of this disease.

Immune-checkpoint blockade has ushered in a new era of cancer immunotherapy with durable responses across several solid tumor types, yet has been disappointing in OC [4]. In comparison to melanoma or lung cancer, OC is typically classified as “immune cold” with low neoantigen load, impaired antigen presentation, high stromal burden, and an overall immunosuppressive tumor microenvironment (TME) [5]. Immune suppression is further driven by tumor-associated macrophages (TAMs) with M2 phenotype, regulatory T cells (Tregs), myeloid-derived suppressor cells (MDSCs) and cytokines such as IL-10 and TGF-β, which collectively prevent activation of effector T cells and promote tumor growth and metastasis [6]. In addition, tumor cells themselves exploit metabolic alterations such as tryptophan depletion via indoleamine 2,3-dioxygenase (IDO), lactate accumulation, and hypoxia-induced changes, which also impair antitumor immunity [7]. However, tumor immune evasion is a major contributing factor to the limited efficacy of first-generation immunotherapies, including PD-1/PD-L1 inhibitors observed in ovarian cancer clinical trials.

Next-generation immunotherapies that go beyond first-generation, single-agent immune checkpoint blockade and instead utilize mechanistically informed combinations, engineered immune-cell platforms, or delivery/tumor microenvironment-modulating technologies. Selected approaches that fall into this category and have demonstrated preclinical or clinical activity against ovarian cancer include multi-checkpoint inhibition, CAR-T and CAR-NK cells, bispecific immune engagers, personalized neoantigen vaccination, oncolytic viruses, nanoparticle-mediated immunomodulation, and biomarker-directed precision immunotherapy [8]. Advances in cancer vaccines, adoptive cell therapies (ACT) including CAR-T and CAR-NK cells, bispecific antibodies, oncolytic virus platforms, and nanoparticle-based immune modulation are expanding therapeutic options [9]. These novel approaches are designed to increase tumor immunogenicity, enhance cytotoxic immune responses and overcome immunosuppressive signals within the OC TME [10]. A recent multi-omics study in ovarian cancer used comprehensive integration of transcriptomic and spatial transcriptomics data to characterize metabolic and immune interaction features that drive tumor progression and immune evasion, identifying candidate pathways and cell–cell communication networks that may serve as therapeutic targets [11]. In this review, we summarize the current understanding of molecular and cellular mechanisms of immune evasion in ovarian cancer and highlight the most promising next-generation immunotherapy approaches currently being explored in preclinical and clinical development. Additionally, we also provide a forward-looking perspective on the future of immunotherapy in the management of OC by examining how emerging therapies may be combined to overcome resistance mechanisms and improve durable responses.

## 2. Molecular Mechanisms of Immune Evasion in Ovarian Cancer

### 2.1. Defective Antigen Presentation

One of the main mechanisms that ovarian cancer uses to avoid immune detection and to decrease activation of cytotoxic T cells is impaired antigen presentation. Tumor cells have downregulated expression of major histocompatibility complex class I (MHC-I) molecules, which present tumor antigens to CD8^+^ T lymphocytes [12]. A decrease in MHC-I expression hinders priming of these T cells and limits the success of immunological checkpoint blockade [13]. Loss or mutation of β2-microglobulin, which is also part of the MHC-I complex, results in an additional defect in antigen presentation [14]. This causes impaired peptide loading and surface expression of MHC-I molecules due to their decreased stability, which essentially makes these tumor cells invisible to cytotoxic T lymphocytes and able to expand in the presence of immune pressure [15].

Neoantigen load in ovarian tumors is relatively low due to the low mutational rate of these tumors when compared with highly immunogenic tumors such as melanoma [16].

Low-grade serous ovarian carcinoma, by comparison, often has lower mutational burden and neoantigen load overall, often harbors KRAS or BRAF driver mutations, and tends to be more immunologically “cold”. Clear cell ovarian carcinoma is another unique subtype that often experiences ARID1A alterations and possesses distinct metabolic and immune-infiltration characteristics that could lead to differences in immunotherapy sensitivity in certain patients [17]. This low expression of neoantigens results in a decreased likelihood of strong T-cell responses and a narrow repertoire of epitopes available for targeting with vaccines and personalized immunotherapies.

High-grade serous ovarian carcinoma (HGSOC), the most common subtype, harbors TP53 mutations in virtually all cases. HGSOC also experiences high levels of chromosomal instability that are caused by dysregulation of DNA damage repair pathways, such as homologous recombination deficiency [18]. Compared to highly immunogenic tumors like melanoma, ovarian cancers like HGSOC tend to have lower mutational burdens at the single-nucleotide level [19]. However, their genomic instability often leads to structural variants, copy-number mutations, and dysregulated gene fusions that may result in diverse neoantigen profiles that could promote immune recognition in certain contexts, especially when agents that target DNA damage are used.

**Figure 1:**
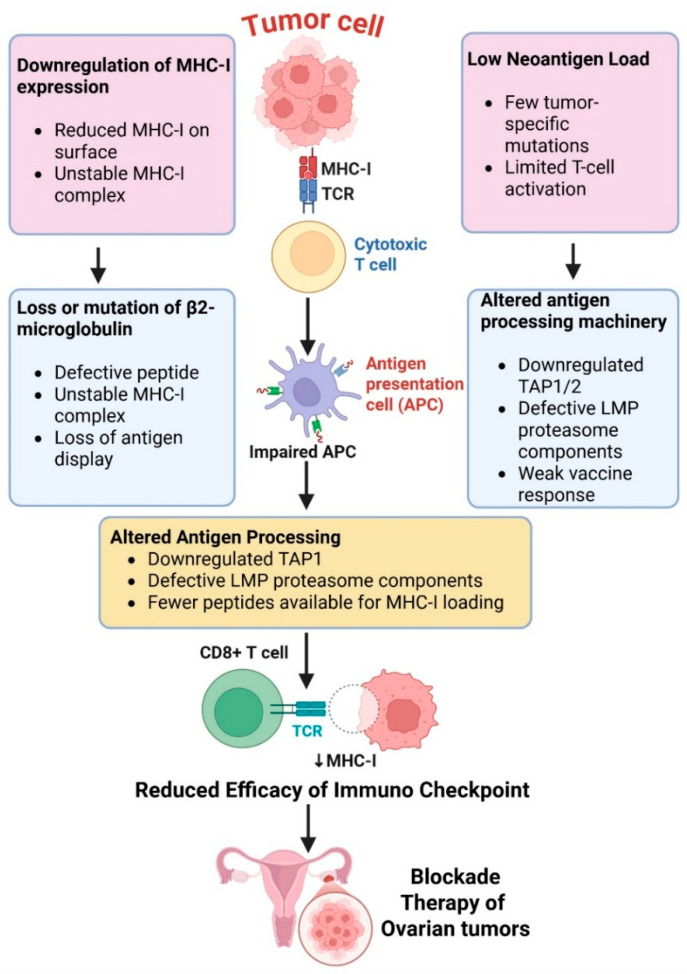
Defective antigen presentation in ovarian cancer. Cancer cells reduce MHC-I expression, resulting in reduced surface MHC-I and unstable antigen–MHC complexes, reducing CD8^+^ T-cell recognition. Insufficient or mutated β2-microglobulin destabilizes MHC-I, leading to ineffective peptide loading and antigen presentation. Ovarian malignancies have less neoantigen load, limiting tumor-specific epitopes for T-cell priming. Peptides for MHC-I loading are also reduced by antigen processing machinery changes, such as TAP1/2 transporter loss or LMP proteasome subunit defects. Defects in the APC phenotype limit CD8^+^ cytotoxic T-cell activation, enhancing immunological resistance to immune checkpoint inhibition in ovarian cancer. The figure was created and modified using BioRender.com.

Ovarian cancer cells can also have alterations in antigen-processing machinery components like TAP1/2 and LMP proteins, further hindering the generation of antigens for presentation [20]. It has been suggested that changes in expression of TAP1/2 and immunoproteasome subunits (LMP2/PSMB9 and LMP7/PSMB8) in ovarian cancer are often caused by epigenetic/transcriptional deregulation rather than permanent genetic loss [21]. Multiple epigenetic mechanisms have been linked to repression of APM expression, including hypermethylation of APM gene promoters, as well as histone modifications limiting transcription of TAP and LMP genes [22]. HDACs and DNMTs have been demonstrated to suppress TAP1/2 and immunoproteasome expression in various solid tumors and ovarian cancer cell lines [23]. Mechanistically, inhibition of HDACs or DNMTs leads to restoration of antigen processing function and MHC-I surface expression that can increase tumor immunogenicity and susceptibility to T-cell-mediated cytolysis [24]. The interferon regulatory factors IRF1 and IRF8, as well as STAT1, are positive transcriptional regulators of APM components [25]. Aberrant IFN-STAT signaling is common in ovarian tumors, leading to defective induction of TAP1/2 and immunoproteasome components. The PI3K/AKT/mTOR pathway and MYC have also been shown to repress IFN-induced transcriptional responses [26]. Overall, these defects contribute to the “cold” immunophenotype of ovarian tumors and make them less susceptible to current immunotherapies (Figure 1).

Immune escape through downregulation of MHC class I expression and β2-microglobulin (β2M) alterations are being appreciated as mechanisms in ovarian cancer with differing frequency between histologic subtypes [27]. Loss of heterozygosity of HLA genes has also been identified in ovarian cancer. In high-grade serous ovarian carcinoma (HGSOC), the most prevalent and aggressive form of ovarian cancer, loss of MHC-I expression is less common than incomplete or heterogeneous loss of expression [28]. Partial MHC-I downregulation identified by IHC or transcriptomic evidence of decreased expression occurs in up to half of HGSOC tumors and has been associated with immunologically “cold” tumors and low CD8^+^ T-cell infiltration. Loss of MHC-I caused by mutations in β2M has also been identified but appears to be less common in ovarian cancer than melanoma or colorectal cancer [29]. β2M mutations have been reported in subsets of HGSOC tumors exhibiting immune selective pressure or tumors that are recurrent. Reduced expression of MHC-I has also been observed in clear cell and endometrioid ovarian carcinomas, which may contribute to these subtypes having different immune landscapes than other forms of ovarian cancer [30]. Clinically, reduced MHC-I and loss of β2M have been associated with reduced progression-free survival and poor response to immune checkpoint inhibitors in ovarian cancer patients.

### 2.2. Immune Checkpoint Activation

**Figure 2:**
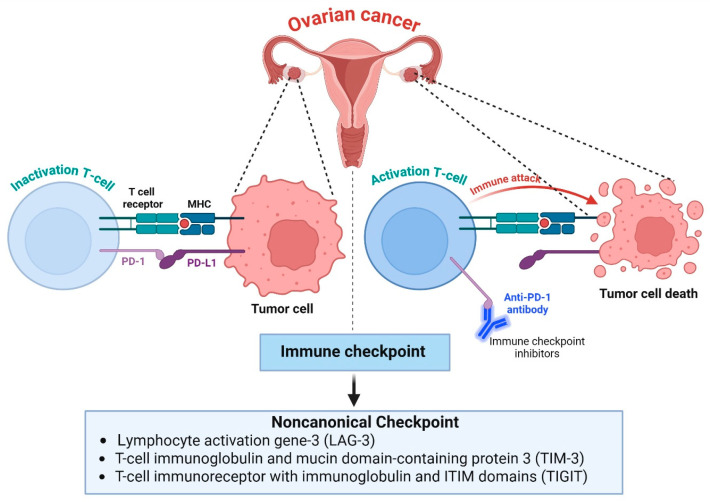
Immune checkpoint activation in ovarian cancer. On the left, tumor-expressed PD-L1 interacts with T cell PD-1 to inactivate, exhaust, reduce cytokine production, and reduce cytotoxic activity. This link lets tumors avoid immune detection. In the central panel, anti-PD-1 antibodies block PD-1/PD-L1 signaling, reactivating T cells. On the right, activated T cells trigger a strong immune response and tumor cell death. In ovarian cancer, noncanonical immunological checkpoints such as LAG-3, TIM-3, and TIGIT facilitate T-cell dysfunction and immune evasion. Cancer-infiltrating cells express these inhibitory receptors, which increases exhaustion and emphasizes the need for combined checkpoint inhibition to improve therapy efficacy. The figure template was modified and created using BioRender.com.

The induction of immunological checkpoints is another mechanism of immunosuppression that ovarian cancer uses to dampen T-cell activity and promote immune evasion [31]. High PD-1 and PD-L1 expression on tumor-infiltrating immune cells in ovarian tumors, specifically those with a high inflammatory infiltrate and/or high-grade histology, is a frequent event [32]. T cell PD-1 engagement with tumor-expressed PD-L1 inhibits T-cell activity, promoting T-cell exhaustion, decreased cytokine secretion, and cytotoxicity [33]. CTLA-4 binds to B7 with a higher affinity than CD28 and suppresses early T-cell activation, thus creating a tolerogenic immune environment. In addition to these canonical checkpoints, ovarian cancer also upregulates several noncanonical inhibitory pathways that further limit antitumor immunity. These include lymphocyte activation gene-3 (LAG-3), T-cell immunoglobulin and mucin domain-containing protein 3 (TIM-3), and T-cell immunoreceptor with immunoglobulin and ITIM domains (TIGIT) [34]. Co-expression of two or more of these checkpoints on tumor-infiltrating lymphocytes is associated with a state of deep exhaustion and predicts a poor response to single-agent checkpoint blockade therapy (Figure 2). LAG-3 and other inhibitory receptors are commonly co-expressed with PD-1 on exhausted tumor-infiltrating lymphocytes in ovarian cancer and are upregulated specifically in tumors that have become resistant to blockade of PD-1/PD-L1 signaling [35]. Therefore, blockade of dual or multiple checkpoints may have advantages over single-agent therapies. However, ovarian cancer has marked inter- and intra-tumoral heterogeneity [36]. Furthermore, mechanisms of immune resistance extend beyond checkpoint blockade and include antigen presentation defects, metabolic disruption, stromal exclusion, and immunosuppressive myeloid cells. For these reasons, LAG-3 and other new checkpoints should be considered complementary targets of opportunity. They are likely to have their greatest impact in rational combination regimens, whether that be with other checkpoint inhibitors like PD-1, tumor microenvironment-modifying agents, or agents that promote tumor immunogenicity. Synergy between these inhibitory pathways may allow ovarian cancers to use redundant immunosuppressive mechanisms to blunt the effects of immune checkpoint inhibition [37]. As such, combination therapies targeting multiple checkpoints may have the potential to reverse T-cell dysfunction and improve clinical outcomes in ovarian cancer.

### 2.3. Immunosuppressive Tumor Microenvironment

**Figure 3:**
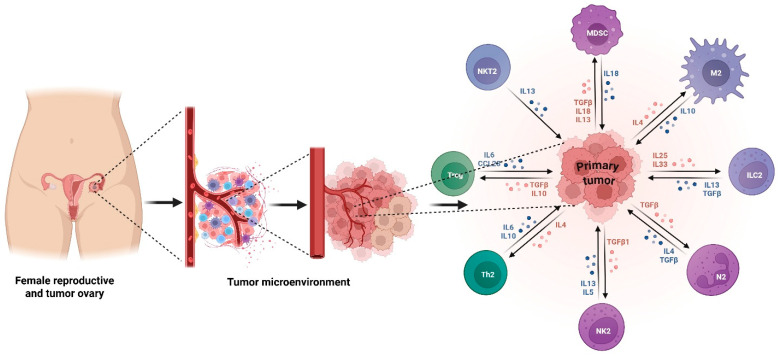
Immunosuppressive tumor microenvironment in ovarian cancer. The primary tumors attract regulatory T cells (Tregs), myeloid-derived suppressor cells (MDSCs), M2-polarized tumor-associated macrophages (TAMs), type 2 innate lymphoid cells (ILC2), NKT2 cells, Th2 cells, and neutrophil subtype N2. These cells generate immunosuppressive cytokines, such as TGF-β, IL-10, IL-4, IL-5, IL-6, IL-13, IL-18, IL-25, and IL-33, which inhibit cytotoxic immune action and promote tumor growth. Tregs and MDSCs prevent effector T-cell proliferation and activation by generating ROS and depriving them of nutrients, while M2 macrophages and Th2-associated cytokines promote angiogenesis, metastasis, and stromal remodeling. CCL28 and IL-6 recruit Tregs and other suppressive cells. The cytokine environment promotes tumor growth, immunological evasion, and immunotherapeutic resistance. Understanding these interactions is essential for developing TME-targeted ovarian cancer treatments. The figure template was modified and created using BioRender.com.

The ovarian cancer TME is highly immunosuppressive and is a key driver of tumor growth and immune escape. Regulatory T cells (Tregs) accumulate in ovarian tumors and ascites, where they block effector T-cell proliferation through secretion of IL-10 and TGF-β and direct cell–cell contact [38]. Their increased infiltration is linked to poor prognosis and decreased treatment response. Myeloid-derived suppressor cells (MDSCs) suppress T-cell activation through arginase-1-mediated L-arginine depletion, inducible nitric oxide synthase (iNOS) activity, reactive oxygen species production, and disruption of T-cell receptor signaling [39]. High arginase 1 expression by MDSCs causes extracellular L-arginine depletion and decreased expression of the CD3ζ chain of the T-cell receptor complex. There is also inhibition of T-cell proliferation and activation [40]. Tumor-associated macrophages (TAMs) compose a significant proportion of immune cells found in the ovarian cancer tumor microenvironment. They have largely an immunosuppressive M2-like phenotype and promote tumor progression, angiogenesis, metastasis, and therapy resistance [41]. M2 TAMs secrete IL-10, VEGF, and chemokines that recruit more immunosuppressive immune cells [42], thus creating a microenvironment favorable to tumor persistence. The chemokine network CCL22-CCR4 recruits Tregs, while CXCL12 maintains an immunosuppressive stromal barrier that blocks the infiltration of effector T cells (Figure 3). Taken together, these elements and interactions build an environment that strongly suppresses immune surveillance. Tumor microenvironment reprogramming, including tumor-associated macrophage repolarization, myeloid-derived suppressor cell inhibition, and regulatory T-cell depletion, is one potential strategy to improve the effectiveness of immunotherapy in ovarian cancer.

### 2.4. Metabolic Reprogramming

Metabolic reprogramming in ovarian cancer can lead to an altered tumor microenvironment and prevention of effective antitumor immunity. As ovarian cancers are associated with enhanced glycolytic flux, they can result in a lactate-rich microenvironment with a reduced pH, which can prevent the activity of cytotoxic T cells and NK cells. High levels of lactate also lead to the promotion of M2 macrophage polarization, the inhibition of dendritic cell maturation, and the activation of regulatory T cells (Tregs), which lead to increased immune tolerance [43]. The overexpression of indoleamine 2,3-dioxygenase (IDO) leads to the depletion of tryptophan and the overproduction of immunosuppressive metabolites like kynurenine, which prevents T-cell proliferation and activation, leading to T-cell exhaustion [44]. The depletion of L-arginine due to arginase also prevents T-cell receptor signaling and effector cell activity.

The glutamine addiction seen in ovarian cancer metabolism can lead to rapid tumor proliferation, as well as indirectly preventing antitumor immunity through the deprivation of glutamine needed for activated immune cells [45]. The hypoxic nature of advanced ovarian cancers also results in the stabilization of HIF-1α, which controls the regulation of angiogenesis, glycolysis, and immunosuppressive proteins like PD-L1 [46]. Systematic profiling of the ovarian cancer tumor microenvironment during hypoxia revealed that hypoxia is associated with increased infiltration of Tregs and immature dendritic cells and that hypoxic signaling driven by HIF-1α leads to recruitment of immunosuppressive immune subsets [47]. Overall, these metabolic changes help create an immunosuppressive microenvironment that leads to immune evasion. The targeting of metabolic checkpoints like IDO, lactate transporters, and hypoxia pathways is a potential therapeutic strategy to restore immunological function and improve immunotherapy response.

### 2.5. NK Cell Exhaustion and Dysfunction

Natural killer (NK) cells have a crucial role in antitumor innate immune defense. However, in ovarian cancer, NK cytotoxic function is significantly impaired by depletion and dysfunction. Ovarian cancers downregulate activating NK-cell receptors (e.g., NKG2D and NKp30) and upregulate inhibitory ligands (e.g., HLA-E and HLA-G) that bind to inhibitory NK receptors (e.g., NKG2A and KIRs) [48,49]. The relative overexpression of inhibitory ligands in ovarian cancers puts NK cells in an inhibited state with lower levels of cytotoxic degranulation and cytokine production. Chronic exposure to immunosuppressive cytokines such as TGF-β also severely impairs NK-cell activity through reduced production of granzyme and perforin and a decrease in metabolic fitness required for cytotoxic responses [50].

Tumor-associated ascites, which is abundant in advanced ovarian cancer, is rich in soluble NKG2D ligands that induce loss of the receptor and functional depletion of NK cells. Hypoxia also skews NK-cell development and impairs their ability to mediate antibody-dependent cellular cytotoxicity [51]. The interaction between NK cells and immunosuppressive populations (e.g., MDSCs) further potentiates NK cell failure. Therefore, ovarian cancers successfully evade innate immune defenses. Reinvigorating NK-cell function with adoptive NK-cell transfer, cytokine priming, blockade of inhibitory checkpoint receptors (such as NKG2A), or nanoparticles to deliver activating signals is a promising next-generation therapeutic strategy [52].

## 3. Next-Generation Immunotherapies

A comparative evaluation of next-generation immunotherapy modalities, including ICIs, adoptive cell therapies, vaccines, immune engagers, oncolytic viruses, and nanomedicine-based platforms, is summarized in Table 1 to highlight their distinct advantages, limitations, and translational barriers in ovarian cancer.

### 3.1. Immune Checkpoint Inhibitors (ICIs)

Immune checkpoint inhibitors (ICIs) are a major class of drugs in modern oncology; however, their response in ovarian cancer has been limited. Clinical trials of anti-PD-1 and anti-PD-L1 agents such as nivolumab, pembrolizumab, and atezolizumab have shown modest response rates, often in the range of 8–15% [53,54,55]. The limited success has been due to the immunologically “cold” nature of ovarian cancers, which often have poor T-cell infiltration, low neoantigen load, and a highly suppressive tumor microenvironment. Thus, single-agent immune checkpoint inhibitors have had little sustained success. Combination checkpoint blockade strategies are being vigorously investigated to overcome these limitations. The combination of PD-1 and CTLA-4 blockade aims to enhance early T-cell activation and to “reinvigorate” exhausted T cells, and combinations with newer checkpoints such as LAG-3 offer additional means to overcome states of deep exhaustion [56]. Preliminary studies with PD-1 and LAG-3 inhibitors have demonstrated synergistic T-cell activation in preclinical models of ovarian cancer [57]. Another promising strategy involves attempts to convert cool tumors into hot tumors to increase response to immune checkpoint inhibitors. This includes using chemotherapy, PARP inhibitors, antiangiogenic therapy, radiation, and oncolytic viruses to increase antigen presentation, improve T-cell infiltration, and alter the tumor microenvironment [58]. Combination strategies may be key to unlocking the full potential of ICIs in ovarian cancer.

### 3.2. Adoptive Cell Therapies

Adoptive Cell Therapies (ACTs) have also been identified as a potential next-generation immunotherapy approach in ovarian cancer using genetically modified or expanded cytotoxic immune cells. CAR-T approaches targeting MUC16, mesothelin and FRα, demonstrating promising preclinical activity, have begun entering early phase clinical development in ovarian cancer [59]. This includes early Phase I trials investigating MUC16-directed CAR-T cells (i.e., NCT02498912), whose primary outcomes were demonstrating feasibility and safety with few objective responses but some evidence of transient disease stabilization in heavily pretreated patients with ovarian cancer. Data from available mesothelin-directed CAR-T trials (i.e., NCT01583686, NCT02159716) are also included and demonstrate manageable safety profiles with limited antitumor activity attributed to limited persistence and infiltration of CAR-T cells into the tumor. Clinical translation has been hampered, however, by tumor antigen heterogeneity, poor trafficking and persistence of CAR-T cells within the immunosuppressive TME, as well as safety concerns surrounding on-target/off-tumor toxicity and cytokine release syndrome [60]. CAR-NK cell therapies are being developed as off-the-shelf cell therapy alternatives to CAR-T cells, which may also offer several benefits, including diminished CRS and neurotoxicity, lack of GVHD, and CAR-independent tumor killing through innate NK-cell mechanisms [61]. Challenges remain, including in vivo persistence and expansion. Regarding CAR-NK therapies, early clinical trials are still underway (for example, NCT03940833), with a focus on promising safety profiles and decreased cytokine release syndrome, but there is preliminary evidence of cytotoxicity, though strong efficacy results are pending [62]. CAR-NK approaches directed against mesothelin and FRα have shown enhanced cytotoxicity and improved safety profiles when compared to CAR-T-cell platforms [63]. TIL therapy represents a promising therapeutic strategy in ovarian cancer, which uses endogenously existing T cells with the ability to recognize tumor antigens. Expanded ex vivo TILs that have been reinfused have shown durable responses in melanoma and are beginning to show early promise in ovarian cancers with high levels of neoantigens or T-cell infiltration [64]. Advancements in TIL expansion, combinatorial therapies and biomarker discovery could help improve the efficacy of ACT in ovarian cancer.

**Table 1:** Comparative overview of next-generation immunotherapies in ovarian cancer.

Therapeutic Strategy	Key Parameters	Mechanism of Action	Clinical Maturity	Efficacy Signals	Toxicity Concerns	Major Limitations	Translational Barriers	Ref
**Immune Checkpoint Inhibitors (ICIs)**	PD-1, PD-L1, CTLA-4, LAG-3	Restore exhausted T-cell function by blocking inhibitory checkpoint signaling	Phase I–III	Modest ORR (≈8–15%); improved responses in combination regimens	Immune-related adverse events, autoimmunity	Low efficacy as monotherapy in “cold” tumors	Poor patient stratification, low neoantigen burden, immunosuppressive TME	[65]
**CAR-T-Cell Therapy**	MUC16 (CA125), FRα, Mesothelin	Engineered T cells recognize tumor antigens and mediate direct cytotoxicity	Early Phase I–II	Strong preclinical activity; limited durable clinical responses	Cytokine release syndrome (CRS), neurotoxicity, on-target/off-tumor toxicity	Antigen heterogeneity, limited persistence, TME suppression	Manufacturing complexity, safety concerns, poor tumor infiltration	[59]
**CAR-NK-Cell Therapy**	Mesothelin, FRα	CAR-mediated killing plus innate NK-cell cytotoxicity	Preclinical–Early Phase I	Enhanced safety, promising cytotoxicity	Lower CRS risk compared to CAR-T	Short lifespan, limited in vivo persistence	Scaling, durability, optimization of cytokine support	[66]
**Tumor-Infiltrating Lymphocytes (TILs)**	Endogenous tumor-reactive T cells	Expansion and reinfusion of autologous tumor-specific T cells	Early clinical exploration	Durable responses in selected patients	Lymphodepletion-related toxicities	Requires high neoantigen load or pre-existing immunity	Limited applicability, labor-intensive expansion	[67]
**Cancer Vaccines (Neoantigen, DC, Peptide, mRNA)**	Tumor-specific neoantigens, TAAs	Enhance antigen presentation and induce tumor-specific T-cell responses	Phase I–II	Robust immune activation, variable clinical benefit	Generally, well tolerated	Insufficient immunogenicity alone	Need combination therapy, antigen selection challenges	[68]
**BiTEs/TriKEs**	MUC16, EpCAM, Mesothelin, CD3, IL-15	Redirect T cells or NK cells to tumor cells via immune synapse	Preclinical–Early Phase I	Strong preclinical tumor lysis	CRS, off-tumor toxicity	Poor penetration in solid tumors	Antigen heterogeneity, short half-life	[69]
**Oncolytic Viral Therapy**	Adenovirus, HSV, Vaccinia	Selective tumor lysis and induction of immunogenic cell death	Phase I–II	Tumor regression in preclinical models	Generally, well tolerated	Limited single-agent durability	Delivery, antiviral immunity, tumor penetration	[70]
**Nanoparticle/Biomaterial-Assisted Immunotherapy**	Antigens, adjuvants, cytokines, siRNA	Targeted delivery and TME modulation	Preclinical–Early translational	Enhanced immune activation in models	Platform-dependent toxicity	Complex formulation	Regulatory hurdles, scalability	[71]

### 3.3. Cancer Vaccines

Cancer vaccines are designed to induce long-term antitumor immunity by augmenting tumor antigen presentation and the robust activation of T cells. Neoantigen vaccines are a relatively novel strategy in ovarian cancer, which leverages mutations present in a patient’s tumor to produce highly immunogenic epitopes that can induce a specific CD8^+^ and CD4^+^ T-cell response [72]. Improvements in sequencing technologies have enabled rapid neoantigen discovery and the development of personalized vaccines that are not limited by antigen presentation constraints, as with traditional vaccines. Dendritic cell vaccines are an attractive approach, as they can augment the efficiency of antigen presentation. Dendritic cells pulsed with tumor lysate, peptides, or mRNA in the setting of ovarian cancer have been able to induce the expansion of tumor-specific T lymphocytes and decrease tumor burden in early-phase trials [73]. These vaccines are particularly attractive when combined with checkpoint inhibitors, which can amplify the T-cell response generated by the vaccine.

Peptide-based and mRNA vaccine technologies have also become more popular given their safety, scalability, and ability to be programmed with a variety of antigenic targets [74]. mRNA vaccines have become an attractive vaccine platform given the successes seen in the COVID-19 vaccine, which have translated to rapid production and high immunogenicity that are attractive for the development of personalized cancer vaccines [75]. While response rates vary, their combination with immune checkpoint inhibitors, PARP inhibitors, or drugs that modulate the tumor microenvironment may dramatically improve vaccine efficacy in ovarian cancer.

### 3.4. Bispecific and Trispecific T-Cell Engagers (BiTEs, TriKEs)

Bispecific T-cell engagers (BiTEs) and Tri-specific killer engagers (TriKEs) are a class of immunotherapeutics that direct cytotoxic immune cells to tumor targets [76]. BiTEs bridge between a tumor-associated antigen and CD3 on T cells, thus leading to formation of an “immune synapse” and to efficient lysis of the target tumor cell [77]. TriKEs are similar in function but have an added domain, generally IL-15, which is intended to improve NK-cell proliferation and persistence. These strategies improve efficacy while lowering the chance for immune escape. This strategy has seen great success in hematologic malignancies, most notably blinatumomab for acute lymphoblastic leukemia, and has shown to be a proof of concept for T-cell redirecting therapies [78]. Translating this success to ovarian cancer is currently an area of active research. BiTEs targeting MUC16, EpCAM, and mesothelin have shown potent antitumor effects in preclinical models of tumor eradication. Safety, PK, and potential clinical activity are now being determined in early-phase clinical trials in recurrent ovarian cancer [79]. However, challenges remain, including antigen heterogeneity, off-tumor toxicity, cytokine release syndrome and poor infiltration of immune cells into solid tumors. BiTEs may find an improved therapeutic index when combined with ICIs or TME modulators or are embedded in nanocarriers for drug delivery [80]. Advances in engineering techniques have enabled the development of next-generation BiTEs and TriKEs that show great promise in revolutionizing ovarian cancer immunotherapy.

### 3.5. Oncolytic Viral Immunotherapy

**Figure 4:**
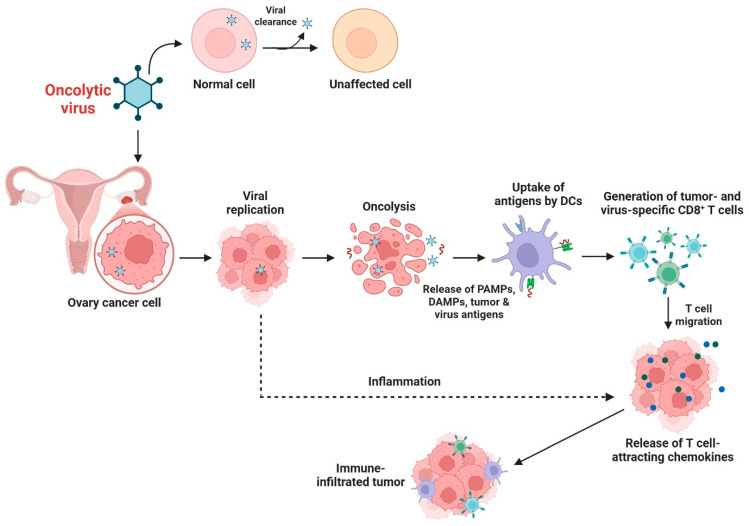
Oncolytic viral immunotherapy in ovarian cancer. To minimize tissue damage, oncolytic viruses replicate in ovarian cancer cells and destroy them, while normal cells are cleaned of viruses. Oncolysis and the production of PAMPs, DAMPs, tumor antigens, and viral antigens are linked to viral replication. Signals are identified by dendritic cells, leading to antigen processing, presentation, and CD8^+^ T-cell generation for tumors and viruses. In response to inflammatory signals and T cell-attracting chemokines, cytotoxic T cells travel to the tumor, enhancing immune cell infiltration. Thus, “cold” ovarian malignancies with little immune infiltration can be made “hot” and more susceptible to anticancer immune activation, improving immunotherapy. The figure template was modified and created using BioRender.com.

Oncolytic viral immunotherapy involves the use of naturally occurring or genetically engineered viruses that preferentially kill tumor cells and stimulate antitumor immunity. Ovarian cancer is highly susceptible to oncolytic viral therapy, given its accessible peritoneal metastases and immunosuppressive tumor microenvironment [81]. Oncolytic adenovirus, HSV, and vaccinia virus-based platforms have been most thoroughly studied in preclinical and clinical settings [82]. These oncolytic viruses replicate in tumor cells, induce cell death, and release tumor antigens that can boost dendritic cell activation and T-cell priming. Oncolytic viruses can induce immunogenic cell death, increase antigenicity and improve tumor sensitivity to immune checkpoint inhibitors [83]. The overexpression of immuno-stimulatory cytokines such as GM-CSF, IL-12, or co-stimulatory ligands can further enhance local immune activation and the recruitment of cytotoxic effectors [84]. In ovarian cancer models, local administration of oncolytic viruses has resulted in significant tumor regression and prolonged survival. Preclinical and clinical trials with oncolytic HSV (T-VEC), adenoviruses, and vaccinia-based vectors have found that this therapy is well-tolerated, yet clinical efficacy remains modest [85]. Combinations of oncolytic viral therapy with immune checkpoint inhibitors, CAR-T cells, or cancer vaccines are a promising approach to boost antitumor immunity and overcome immunotherapy resistance in ovarian cancer [86]. The mechanism of action of oncolytic viral immunotherapy in ovarian cancer details is presented in Figure 4.

### 3.6. Nanoparticle and Biomaterial-Assisted Immunotherapy

Nanoparticle and biomaterial-assisted immunotherapies have shown great promise in boosting immune activation and overcoming challenges associated with traditional immunotherapy approaches in ovarian cancer. Nanocarriers can effectively deliver tumor antigens, adjuvants, cytokines, or immunomodulatory agents directly to the tumor microenvironment, thereby increasing efficacy and reducing systemic toxicity [87]. Nanoparticle-based vaccines allow for sustained release of antigenic materials and improved uptake by dendritic cells, resulting in stronger and more sustained T-cell responses [88]. Liposomes, polymeric nanoparticles, dendrimers, and lipid nanoparticles have all demonstrated potential in co-delivering antigens and immune stimulants that promote anticancer immunity [89]. One of the most exciting aspects of nanomedicine is its potential to modulate the tumor microenvironment. Nanoparticles designed to reprogram tumor-associated macrophages from an M2 to an M1 phenotype can restore immune surveillance and increase responsiveness to immunotherapy [90]. Similarly, nanoparticles delivering IDO inhibitors, siRNA, or metabolic modulators can disrupt immunosuppressive networks and restore T-cell function in ovarian tumors [91]. Biomaterial scaffolds and hydrogels can enhance the delivery of CAR-T or CAR-NK cells and improve their persistence and resistance to hostile tumor microenvironment conditions. These platforms also provide continuous cytokine support and customizable activation signals that can further boost treatment efficacy. With continued advancements in engineering technologies, nanomedicine-based immunotherapies represent a critical avenue for developing durable and effective next-generation therapies for ovarian cancer.

## 4. Targeting the Tumor Microenvironment

### 4.1. TAM Reprogramming (CSF1R, CD47-SIRPα Axis)

Tumor-associated macrophages (TAMs) represent the predominant immune cell infiltrate in the tumor microenvironment of ovarian cancer and have been shown to be skewed towards M2-like polarization states [92]. M2-like TAMs promote tumor growth, angiogenesis, matrix remodeling, and metastasis while inhibiting antitumor immunity through production of immunosuppressive cytokines like IL-10 and TGF-β. M2-like TAM enrichment has been associated with late-stage disease, ascites accumulation, exclusion of cytotoxic CD8^+^ T cells from the tumor microenvironment, and decreased survival in ovarian cancer. Therapies targeting TAMs aim to either deplete them or reprogram them into a pro-inflammatory M1 phenotype. Drugs targeting the colony-stimulating factor 1 receptor (CSF1R) have shown promise in reducing the recruitment and survival of M2 macrophages [93]. By blocking CSF1-CSF1R signaling, these agents decrease TAM density and increase T-cell infiltration, thus potentiating immunotherapy.

The CD47-SIRPα axis is another “don’t-eat-me” signal that tumor cells use to avoid macrophage phagocytosis. CD47 is highly upregulated in ovarian cancer, and its expression is associated with poor prognosis. Therapeutic antibodies targeting CD47 or SIRPα can restore macrophage phagocytic activity and induce subsequent adaptive immune responses through antigen cross-presentation [94]. Preclinical evidence suggests that the inhibition of CD47 may have synergistic benefits when combined with checkpoint inhibitors or CAR-T-cell therapy [95]. TAM-reprogramming strategies represent a promising way to alleviate immune suppression and improve anti-cancer responses in ovarian cancer.

### 4.2. Cancer-Associated Fibroblasts and Extracellular Matrix Remodeling

Cancer-associated fibroblasts (CAFs) are critical contributors to tumor immune evasion mechanisms, therapy resistance, and metastasis. Ovarian cancer CAFs drive extracellular matrix remodeling with overproduction and crosslinking of collagen, fibronectin, and proteoglycans, causing increased stiffness and a dense desmoplastic stroma [96]. The resulting fibrotic tissue physically prevents cytotoxic immune cells, such as CD8^+^ T cells and NK cells, from infiltrating tumor parenchyma and prevents delivery of chemotherapy and immunotherapy drugs throughout the tumor. In addition, ECM proteins produced by CAFs can bind growth factors and chemokines and create a barrier to immune-cell infiltration [97]. This dense fibrotic stroma mediated by CAFs allows ovarian cancer to thrive within an immunosuppressive environment and be less responsive to drugs [98]. The secretion of CXCL12, TGF-β, and IL-6 can also augment immunosuppression by recruiting regulatory T cells and myeloid-derived suppressor cells and maintaining tumor-associated macrophages in an M2 phenotype [99]. Additionally, CAFs can directly support tumor invasion and peritoneal dissemination through matrix metalloproteinase (MMP) secretion, which degrades ECM structures [100]. The targeting of cancer-associated fibroblasts and ECM remodeling has been a strategy to improve immune access in ovarian cancers. Targeting fibroblast activation protein (FAP), which is expressed on CAFs, can lead to the specific ablation of fibroblasts or can be used to deliver cytotoxic payloads to reprogram the stromal environment [101]. TGF-β signaling inhibitors can also reduce fibrosis and restore ECM architecture while promoting T-cell infiltration. The enzymatic degradation of ECM components, as demonstrated with hyaluronidase therapy, has been shown to enhance antitumor immunity and facilitate the delivery of chemotherapy and immunotherapy agents [102]. These approaches aim to break down CAF-mediated stromal barriers to convert immune-excluded ovarian cancers into more immunologically accessible states.

### 4.3. Hypoxia, HIF Regulation, and Angiogenesis Modulation

Hypoxia is a common feature of high-grade ovarian cancer and a major driver of tumorigenesis and immune suppression. Limited blood vessel formation results in a lack of oxygen, which stabilizes hypoxia-inducible transcription factors (HIF-1α and HIF-2α) [103]. HIF-1α and HIF-2α drive transcription of genes that promote angiogenesis, glycolysis, and immunosuppression [104]. HIF-1α upregulates VEGF, a potent angiogenic factor that promotes leaky, dysfunctional blood vessels that further promote hypoxia and limit T-cell infiltration [105]. Hypoxia also upregulates expression of PD-L1 and other inhibitory cytokines such as IL-10, contributing to T-cell exhaustion [106]. In addition, hypoxic conditions also drive macrophages towards an M2 phenotype and limit dendritic cell maturation and function. Therapies targeting hypoxia and HIF signaling aim to normalize the tumor vasculature and restore immune function. Anti-angiogenic agents, such as bevacizumab and tyrosine kinase inhibitors, block VEGF signaling and improve blood vessel structure to allow for better infiltration by immune cells [107]. Direct HIF inhibitors, or drugs targeting downstream metabolic pathways, can reduce immunosuppressive signaling and improve tumor responsiveness to immunotherapy [108]. Combinations of drugs targeting hypoxia with checkpoint inhibitors or cell therapies have shown synergistic activity in preclinical models. Targeting the hypoxic tumor microenvironment is a promising strategy to overcome immune suppression and improve response to immunotherapy in ovarian cancer.

### 4.4. Targeting PI3K/AKT/mTOR to Reverse Immune Suppression

The PI3K/AKT/mTOR pathway is frequently dysregulated in ovarian cancer and contributes to tumor cell survival, metabolic flexibility, and immune escape [109]. PI3K-AKT activation can support proliferation, inhibit apoptosis, and enable chemoresistance. The pathway also reshapes the immune landscape by upregulating PD-L1, downregulating antigen presentation, and upregulating the production of immunosuppressive cytokines such as IL-10 and TGF-β [110]. Hyperactivated mTOR signaling supports metabolic rewiring, which increases aerobic glycolysis and lactate production, which inhibits T-cell function and promotes M2 macrophage polarization [111]. Inhibiting PI3K, AKT, or mTOR pharmacologically has emerged as a promising approach to boost antitumor immunity. PI3K inhibitors, particularly those targeting the PI3K-γ and PI3K-δ isoforms, can reduce MDSC recruitment and reprogram macrophages towards a pro-inflammatory phenotype [112]. AKT inhibitors can restore apoptotic sensitivity and improve antigen presentation, while mTOR inhibitors like rapalogs can decrease the tumor metabolic load and enhance effector T-cell function [113]. Combination strategies of PI3K/AKT/mTOR inhibitors with checkpoint blockade, cancer vaccines, or adoptive cell therapy have shown promising synergistic effects in preclinical models [114]. Targeting this pathway offers a multi-faceted approach to reverse immune suppression and enhance sensitivity to next-generation immunotherapies.

## 5. Predictive Biomarkers and Precision Immunotherapy

**Figure 5:**
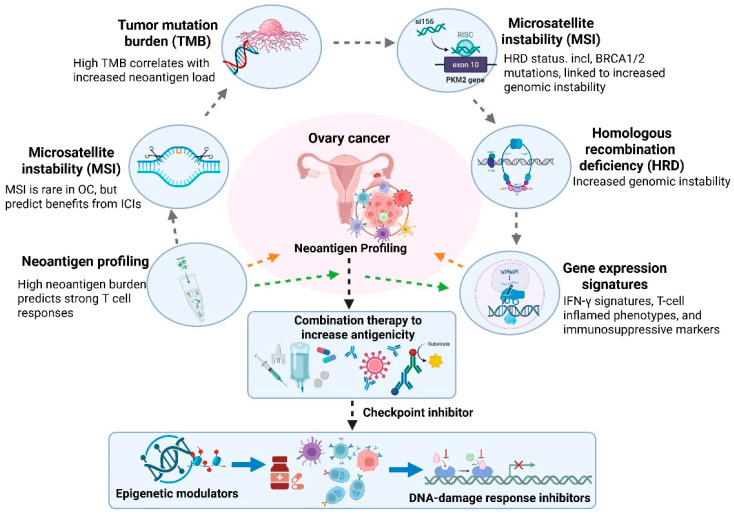
Precision immunotherapy framework in ovarian cancer. Molecular and immunologic biomarkers, such as tumor mutation burden (TMB), microsatellite instability (MSI), HRD/BRCA, neoantigen burden, and gene expression signatures related to immune activation, can help identify immunogenic tumors. High TMB, HRD-associated genomic instability, or neoantigen load tumors may benefit from ICIs, vaccinations, ACT, or TIL therapy. MSI-high tumors, rare in OC, may benefit from checkpoint blocking. Several gene expression characteristics can identify immunologically “hot” vs. “cold” cancers, predict ICI and other therapy response, and guide combinatorial strategies. Epigenetic medicines, DNA-damage response inhibitors, oncolytic viruses, and nanoparticle-based immunotherapies can boost immune checkpoint inhibition and antigenicity. The figure template was modified and created using BioRender.com.

More specifically, MSI-high status (microsatellite instability), while uncommon in ovarian cancer, and HRD/BRCA1/2 alterations are the most clinically validated biomarkers with clear therapeutic implications, including for immune checkpoint inhibitor treatment in specific scenarios and combination PARP inhibitor treatment [115]. Tumor mutational burden (TMB) has been validated as a biomarker in some tumor types but is considered investigational in ovarian cancer and has shown variable predictive ability. Precision immunotherapy leverages molecular, genetic, and immunologic factors to identify patients most likely to benefit from specific interventions. Several biomarkers have received significant interest, including TMB, microsatellite instability (MSI) status, and homologous recombination deficiency (HRD) [116]. While ovarian cancer is typically characterized by low tumor mutational burden (TMB) and a low frequency of MSI, subsets of patients with HRD, such as those with BRCA1/2 mutations, show increased genomic instability [117]. This may increase neoantigen production and may improve responses to immunotherapy in combination with DNA-damage-targeting agents, such as PARP inhibitors [118]. Neoantigen burden is a critical determinant of immunogenicity. Tumors with a high neoantigen burden are more likely to trigger strong T-cell responses, making neoantigen profiling important for the development of personalized cancer vaccines and prediction of checkpoint inhibitor responses [119]. Because ovarian cancer often has low neoantigen levels, strategies to increase antigenicity, including oncolytic viruses, epigenetic modulators, or targeted DNA-damage approaches, are being actively explored. Gene expression signatures, such as those related to interferon gamma (IFN-γ) response, T-cell inflamed signatures, and markers of immunosuppressive pathways, provide additional avenues for patient stratification [120]. These markers can help differentiate cancers with pre-existing immune activation from those that are immunologically “cold” and may require combination therapy to increase immunogenicity (Figure 5). Furthermore, markers of angiogenesis, hypoxia, and stromal activation can be indicative of poor treatment response and guide the inclusion of anti-angiogenic or tumor microenvironment-modulating drugs.

Recent advances in spatial transcriptomics and single-cell sequencing offer unprecedented resolution for understanding tumor–immune dynamics. These technologies allow researchers to map immune cell localization, identify exhausted or dysfunctional T-cell subsets, characterize myeloid suppressor populations, and detect differential expression of immunological checkpoints within tumor microenvironments [121]. These insights will be critical for optimizing combination immunotherapies and understanding resistance mechanisms. Multi-omics approaches (integrating genomes, transcriptomics, epigenomics, proteomics, and metabolomics) are increasingly being used to build comprehensive patient-specific immune profiles [122]. These integrative approaches can help differentiate responders from non-responders, uncover new immunotherapy targets, and improve patient selection for clinical trials. By combining multiple layers of data, researchers can improve their understanding of the interplay between tumor biology, immunological makeup, and metabolic pathways in influencing treatment outcomes [123]. Predictive biomarkers will be critical for ushering in an era of precision immunotherapy in ovarian cancer. Their integration into treatment decision-making processes will improve therapy personalization, response rates, and help develop rational combination strategies to overcome immune resistance. As technical and computational approaches continue to mature, biomarker-driven precision oncology will be a critical piece in improving survivorship for ovarian cancer patients.

## 6. Preclinical Models Driving Next-Generation Immunotherapy

**Figure 6:**
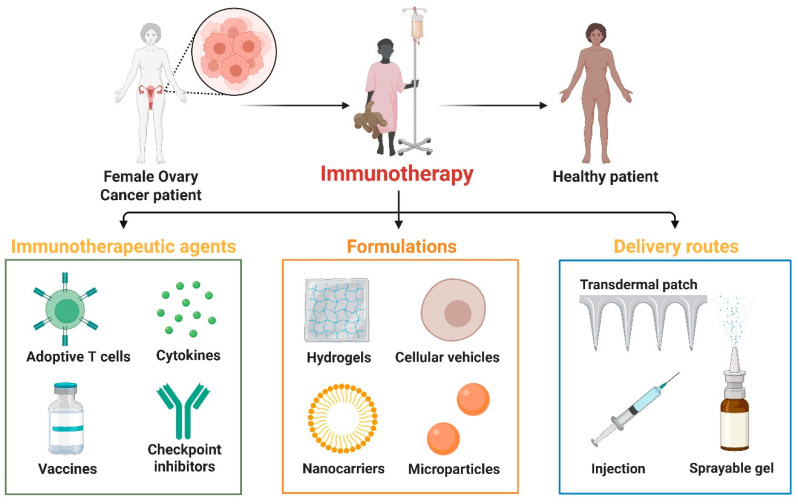
Next-generation immunotherapy in ovarian cancer. Advanced immunotherapies for ovarian cancer and patient recovery. In the upper section, a female ovarian cancer patient becomes well after immunotherapy. The lower panels show three key advanced immunotherapy components. Adoptive T-cell therapies, cytokines, vaccines, and immune checkpoint inhibitors boost anticancer immune responses. Hydrogels, cellular vehicles, nanocarriers, and microparticles stabilize, target, and control immunomodulatory drug release. Transdermal patches, injectable formulations, and sprayable gels provide patient compliance, sustained medication exposure, and optimal therapeutic efficacy. The figure template was modified and created using BioRender.com.

The development of next-generation immunotherapies for ovarian cancer relies heavily on the availability of advanced preclinical models that closely resemble human tumor biology and immunological interactions [124]. Advanced immunotherapy strategies for ovarian cancer, highlighting key therapeutic components (adoptive T cells, cytokines, vaccines, checkpoint inhibitors), complex formulations (hydrogels, cellular vehicles, nanocarriers, microparticles), and delivery systems (transdermal patches, injections, sprayable gels), are designed to enhance immune activation, targeted delivery, and patient recovery [125] (Figure 6).

Traditional two-dimensional (2D) culture systems fail to recapitulate the complex tumor microenvironment (TME), leading to poor translational success [126]. Therefore, platforms such as three-dimensional (3D) spheroids, organoids, patient-derived xenografts (PDX), humanized mouse models, and organoid–immune cell co-culture systems have emerged as critical tools for evaluating immunotherapy responses and resistance mechanisms [127]. Three-dimensional spheroids and organoids faithfully recapitulate the architecture, cellular composition, nutrient gradients, and drug penetration profiles of solid tumors [128]. Ovarian cancer spheroids, like peritoneal metastatic aggregates found in ascites, can be used to study immune infiltration kinetics and treatment resistance [129]. Patient-derived organoids (PDOs) maintain the genomic, epigenomic, and phenotypic features of the original tumor, allowing for personalized medication screening and assessment of immunotherapeutic combinations, such as checkpoint inhibitors, oncolytic viruses, and nanoparticle-delivered immunomodulators [130].

Patient-derived xenograft (PDX) models are a powerful tool, as they maintain tumor heterogeneity and more accurately mimic human disease progression compared to traditional cell-line models [131]. In ovarian cancer, intraperitoneal implantation of patient-derived xenografts models metastatic spread, ascites formation, and chemoresistance [132]. However, conventional PDX models are limited in their ability to evaluate immunotherapies due to the lack of human immune cells. Humanized PDX models overcome this limitation by transplanting human hematopoietic stem cells (HSCs) or peripheral blood mononuclear cells (PBMCs) into immunocompromised mice, reconstituting a functional human immune system [133]. Humanized PDX models allow for accurate assessment of immune checkpoint inhibitors, chimeric antigen receptor (CAR)-T and CAR-natural killer (NK) cells, vaccinations, and novel immunomodulators in a physiologically relevant setting [134].

Organoid–immune cell co-culture systems. Organoid–immune cell co-culture systems represent a major advancement in studying tumor–immune interactions with high fidelity. By introducing autologous T cells, NK cells, macrophages, or dendritic cells into patient-derived organoids, researchers can monitor cytotoxic responses, immune evasion mechanisms, cytokine signaling, and antigen presentation in real time [135]. These technologies are invaluable for identifying biomarkers of immune reactivity, optimizing cell-based therapies, and testing combination regimens that target the TME.

These advanced preclinical models accelerate the translation of next-generation immunotherapies by providing more precise, patient-relevant platforms that capture the complexity of ovarian cancer biology. Their integration into drug development pipelines enhances prediction power, reduces clinical trial attrition rates, and enables precision immunotherapy strategies designed for individual patients. As these models continue to evolve, they will play an increasingly critical role in the design and validation of curative immunotherapies for ovarian cancer.

## 7. Clinical Trials Landscape

To clarify the translational maturity of each therapeutic strategy, Table 2 summarizes the current in vitro, preclinical, and clinical evidence supporting next-generation immunotherapies for ovarian cancer. Selected clinical trials of next-generation immunotherapy for ovarian cancer have shown promise but also highlight ongoing challenges. In KEYNOTE-100 (Phase II study of pembrolizumab monotherapy in advanced recurrent ovarian cancer), the ORR was about 8%, and the disease control rate was about 22% overall across both cohorts [65]. The investigators of a similar study reported similar results showing limited single-agent activity with an ORR (~8%) for PD-1 blockade in the recurrent ovarian cancer setting without agents to combine. Results were reported for the NRG-GY003 trial (randomized Phase II study of nivolumab +/− ipilimumab in recurrent or persistent epithelial ovarian cancer) [54]. At 6 months, objective response rates were 12.2% for nivolumab alone and 31.4% for the nivolumab and ipilimumab combination [54]. In the JAVELIN Ovarian 200 Phase III trial, avelumab monotherapy yielded an objective response rate of 9.6% in patients with platinum-resistant or refractory ovarian cancer [136]. Trials combining immune checkpoint inhibitors (ICIs) with chemotherapy or bevacizumab have resulted in marginal improvements but are insufficient to drive durable clinical benefit [137]. Early trials of dendritic cell vaccines, oncolytic viruses, and adoptive cell therapies have shown safety and evidence of immune activation, but only limited and transient antitumor responses.

Ongoing Phase I–III trials focus on more advanced and integrated approaches. This includes combinations of ICIs with PARP inhibitors (e.g., olaparib + durvalumab), anti-angiogenic agents, or DNA-damage response modulators, aiming to increase tumor immunogenicity [147]. CAR-T- and CAR-NK-cell therapies targeting MUC16, mesothelin, and FRα are progressing through early clinical phases, incorporating advanced engineering strategies to improve persistence and reduce toxicity [148]. Neoantigen vaccines, mRNA-based immunotherapies, and bispecific T-cell engagers (BiTEs) are in early-stage trials, reflecting a shift towards personalized and multi-targeted immunotherapeutic strategies [138]. The integration of humanized mouse-guided biomarker discovery into clinical trial design is beginning to improve patient selection. There are many reasons for the limited success of past trials. These include improper selection, lack of adequate biomarkers, tumor heterogeneity, antigen loss, and excessive immunosuppression in the TME. Additionally, many immunotherapies have shown limited penetration into large tumor masses or peritoneal metastases common in ovarian cancer. Safety concerns, such as cytokine release syndrome in adoptive cell therapy, also limit dose escalation and efficacy.

Improving therapeutic efficacy will require biomarker-driven precision approaches that identify patients most likely to respond to specific drugs. Incorporating multi-omics profiling, spatial transcriptomics, and immune phenotyping into early-phase clinical trials will enhance patient selection [149]. Combination strategies that aim to convert cold tumors to immune-responsive phenotypes (e.g., combining ICIs with hypoxia modulators, tumor-associated macrophage reprogramming therapies, or nanoparticle-mediated vaccines) are expected to improve the durability of response [150]. Additionally, local delivery approaches, such as intraperitoneal infusion of CAR-NK cells, BiTEs, or oncolytic viruses, may overcome drug distribution challenges. With ongoing trials moving toward more mechanistically informed, combination-based, and personalized approaches, the next decade is poised to improve outcomes in ovarian cancer immunotherapy.

## 8. Challenges and Future Directions

The translational landscape of next-generation immunotherapies for ovarian cancer is fraught with formidable challenges, with one of the most prominent being the presence of pronounced immunotherapy resistance. Ovarian cancers frequently display a limited neoantigen burden, insufficient effector T-cell infiltration, and robust immunosuppressive signals orchestrated by regulatory T cells, myeloid-derived suppressor cells (MDSCs), and M2 macrophages [151]. Tumor cells leverage various immunological checkpoint pathways, undergo metabolic reprogramming, and adapt to hypoxic conditions to evade immune surveillance [46]. The convergence of these pathways has rendered single-agent immunotherapy strategies ineffective. Overcoming resistance will necessitate combinatorial approaches that enhance antigenicity, promote T-cell infiltration, and reprogram the suppressive tumor microenvironment.

Acquired genetic mechanisms of resistance, including JAK1/2 alterations, impact interferon-γ-mediated antigen presentation and immune responsiveness or defects in downstream STAT signaling [152]. Mechanisms of acquired resistance can occur due to immune selective pressure and lead to acquired or secondary resistance to immune checkpoint blockade. In addition, tumor evolution and selection of clones during treatment can lead to these alterations, as well as loss of target antigens and rewiring of immune signaling pathways. These changes can also occur due to enrichment of immune-evasive tumor subclones that arise during treatment. An immunosuppressive tumor microenvironment, such as spatial heterogeneity of immune infiltration [153], and differential localization of cytotoxic T cells, suppressive myeloid cells, and fibrotic barriers between intratumoral and metastatic lesions can also impact treatment response [154]. Recent technological innovations allow us to capture the complexities of the tumor microenvironment. Techniques such as spatial transcriptomics and multiplex imaging have begun to emerge as powerful tools to understand spatial heterogeneity and guide rational combination approaches. It has been recently approved or emerging for use in ovarian cancer, such as antibody–drug conjugates (ADCs), including folate receptor alpha-targeted therapies. These therapies may potentiate responses to immunotherapy through promotion of immunogenic cell death or modulation of the tumor microenvironment. Clinical trials that are currently underway are evaluating ADCs in combination with immunotherapy.

A promising strategy lies in the development of personalized, multi-modal combination therapies. Immune checkpoint blockade can be strategically combined with DNA-damage response inhibitors, oncolytic viruses, metabolic modulators, and adoptive cell therapies to target tumor heterogeneity and increase treatment efficacy [155]. Personalized neoantigen vaccines, customized CAR-T or CAR-NK therapies, and biomarker-driven drug selection guided by genomic and transcriptomic profiling can further refine the precision of patient-specific approaches [156]. Combination therapies must consider optimal sequencing and dosing to maximize synergy and minimize toxicity.

The convergence of genomics, nanomedicine, and immunotherapy holds immense promise for the future of ovarian cancer treatment. Genomic profiling can enable the determination of homologous recombination deficiency (HRD) status, mutational biomarkers, immunological signatures, and actionable neoantigens that can guide precision immunotherapy decisions [157]. Nanoparticle-based drug delivery systems can enhance drug stability, optimize tumor targeting, and allow for the co-delivery of antigens, adjuvants, cytokines, or small interfering RNAs (siRNAs) to modulate the tumor microenvironment [10]. The integration of nanotechnology and immunotherapy holds potential in augmenting CAR-T cell persistence, reprogramming tumor-associated macrophages, and improving vaccine delivery efficiency [158]. The integration of multi-omics data with artificial intelligence (AI) algorithms will accelerate the identification of biomarkers, guide therapeutic optimization, and enable real-time patient monitoring.

Safety concerns, including cytokine release syndrome (CRS), neurotoxicity, and off-target effects, remain significant challenges in the translation of advanced therapies such as CAR-T cells and bispecific T-cell engagers. Advanced engineering strategies, such as the incorporation of suicide switches, the development of programmable CAR constructs, the use of low-affinity receptors, and localized intraperitoneal delivery approaches, are being explored to mitigate risks. The development of humanized preclinical models can also improve the predictability of toxicity profiles before initiating clinical trials.

The future of ovarian cancer immunotherapy will be shaped by strategic multi-modal combinations, biomarker-guided patient selection, and advanced delivery platforms that enhance precision while minimizing toxicity. Collaborative efforts among immunologists, oncologists, bioengineers, and computational biologists will be instrumental in translating future scientific discoveries into sustainable clinical benefits. With ongoing innovation, next-generation immunotherapy holds the potential to revolutionize long-term outcomes for patients with ovarian cancer.

## 9. Conclusions

Immunotherapy has tremendous potential to improve the outcome of ovarian cancer, which has shown suboptimal response to immune-based interventions in the past. Recent advances in the understanding of immune escape, tumor microenvironment, and interpatient heterogeneity have led to the identification of new therapeutic targets and strategies to overcome therapeutic resistance. Emerging approaches, including a combination of checkpoint blockade, CAR-T and CAR-NK cells, personalized neoantigen vaccines, oncolytic viruses, and nanomedicine-facilitated immunotherapy, are rapidly changing the treatment paradigm. Multi-omics profiling, spatial analysis, and biomarker-guided patient stratification will be important for treatment personalization and enhancing clinical responses. Continued progress in delivery platforms, engineering of immune cells, and reprogramming of the tumor microenvironment will likely lead to the development of durable and synergistic antitumor immunity. In parallel with coordinated scientific, translational, and clinical efforts, next-generation immunotherapy holds the promise of ultimately revolutionizing the long-term prognosis for patients with ovarian cancer.

## Figures and Tables

**Table 2:** Clinical and preclinical evidence landscape of next-generation immunotherapies in ovarian cancer.

Immunotherapy Modality	In Vitro Studies	Animal Models	Early-Phase Clinical Trials (Phase I–II)	Late-Phase Clinical Trials (Phase III)	Key Outcomes	Ref
**Immune Checkpoint Inhibitors (PD-1/PD-L1, CTLA-4)**	✓ Demonstrated T-cell reinvigoration	✓ Enhanced antitumor immunity in xenografts	✓ Modest ORR (<15%) in recurrent ovarian cancer	✓ Limited benefit, no durable OS improvement	Confirms immunologically “cold” tumor phenotype	[138]
**ICI + Chemotherapy/Bevacizumab**	—	✓ Improved immune infiltration	✓ Marginal response improvement	✓ Insufficient durable benefit	Limited clinical impact as combination strategy	[139]
**ICI + PARP Inhibitors (e.g., Olaparib + Durvalumab)**	✓ Increased DNA damage and antigenicity	✓ Enhanced T-cell recruitment	✓ Ongoing trials with early efficacy signals	—	Promising rationale, clinical benefit under evaluation	[140]
**CAR-T-Cell Therapy (MUC16, Mesothelin, FRα)**	✓ Potent tumor cell lysis	✓ Tumor regression, survival benefit	✓ Safety demonstrated, limited persistence	—	Efficacy is limited by TME and antigen heterogeneity	[141]
**CAR-NK-Cell Therapy**	✓ Enhanced cytotoxicity, lower toxicity	✓ Improved safety and infiltration	✓ Early trials ongoing	—	Favorable safety profile, efficacy optimization needed	[142]
**Cancer Vaccines (DC, Neoantigen, mRNA)**	✓ Robust T-cell priming	✓ Reduced tumor burden	✓ Immune activation, limited tumor regression	—	Best suited for combinatorial regimens	[143]
**Bispecific T-cell Engagers (BiTEs)**	✓ Efficient immune synapse formation	✓ Strong antitumor responses	✓ Early-stage trials in recurrent disease	—	Penetration and CRS remain challenges	[144]
**Oncolytic Viral Immunotherapy**	✓ Induces immunogenic cell death	✓ Tumor regression, immune activation	✓ Well-tolerated, modest efficacy	—	Strong synergy with ICIs	[145]
**Nanoparticle-Based Immunotherapy**	✓ Targeted immune modulation	✓ Improved delivery and efficacy	—	—	Primarily preclinical, high translational potential	[146]

✓ = Evidence reported or under active investigation; — = not yet established.

## Data Availability

No new data were created or analyzed in this study.
